# Nutritional Deprivation Index is negatively associated with socio-economic factors in Paraguayan households

**DOI:** 10.1017/jns.2020.13

**Published:** 2020-06-05

**Authors:** Vit Bubak, Matteo Cellamare, Marta Sanabria

**Affiliations:** 1 Instituto Desarrollo, Guido Spano 2575, Asunción, Paraguay; 2 Centro para la Economía y el Desarrollo Humano, Exc. Juan Estigarribia 6221, Luque, Paraguay; 3 National University of Asunción, San Lorenzo, Paraguay

**Keywords:** Dietary diversity, Socio-economic status, Household surveys, Latin America

## Abstract

The present study aimed to examine the nutritional deprivation of Paraguayan households (measured as households' access to diverse diets) and investigate the association between nutritional deprivation and socio-economic characteristics in a large sample. An extension of Alkire–Foster methodology, a technique widely employed in multidimensional poverty measurement, was used to calculate both the incidence and intensity of nutritional deprivation. The resulting Nutritional Deprivation Index allows us to consider minimum food group requirements that vary by food groups as well as by individual characteristics such as age, sex and activity level. Applying the methodology to a nationally representative sample of households from the 2011–2012 Income and Expenditures Household Survey, the study found that about two in every three Paraguayan households (67 %) were inadequately nourished in at least four (of the total of six) food groups. Although no significant differences were found between rural and urban households, the incidence of multi-dimensionally deprived households generally decreased as income increased. Logistic regression results showed that nutritional deprivation decreased as household income and mother's education increased and increased with household size. Our study concludes that the majority of Paraguayan households is significantly nutritionally deprived across most food groups and suggests that strategies are needed to improve their access to diverse diets, especially among its lower- and middle-income segments.

Dietary diversity has been long recognised as a key element of food-based dietary guidelines. The underlying concept is based on the idea that no one food contains all the necessary ingredients and that increasing the variety of foods both across and within food groups is needed to ensure an adequate intake of essential nutrients and to promote good health^(1)^. Indeed, several studies have found a positive relationship between dietary diversity and nutrient adequacy, both in developed and in developing countries^(2,3)^.

Dietary diversity has been commonly evaluated using a simple count of foods or food groups consumed over a given reference period^(4–6)^. However, this approach has several limitations, especially when the dietary diversity is used as a population-level indicator. These limitations include, among others, (1) failing to account for the extent of inadequate food consumption (in other words, using a cut-off approach whereby all individuals who consume fewer than the minimum number of food groups are treated as equally deprived), (2) disregarding the amount of each food group consumed, and (3) neglecting person-specific variations in food requirements.

In this paper, we seek to address these limitations by applying an extended version of a technique widely used in multidimensional poverty measurement, the Alkire–Foster (AF) methodology^(7–9)^. The AF methodology allows us to measure simultaneous deprivations in multiple dimensions using a counting approach; specifically, given the collection of all dimensions achieved by an individual (household), the AF methodology applies a dual cut-off that first translates dimensions into deprivations and then determines whether the individual (household) is deprived in a pre-specified number of dimensions^(10)^. Assuming that dimensions represent the food groups, it is straightforward to see that this methodology allows us to address the first two limitations by accounting for both the number of under-consumed food groups (incidence) and the amount of each food group consumed (intensity).

An extension of the AF methodology, recently proposed by Oldiges^(9)^, and referred to as a Nutritional Deprivation Index (NDI), allows us to account for the third limitation in that it directly considers individual food requirements^(9)^. In particular, the NDI generalises the AF methodology by allowing us to consider minimum food group requirements (cut-off thresholds) that vary from person to person based on their age, sex, occupation, health status and/or other characteristics.

The NDI methodology extends directly to the population level as it can be applied to calculate both the incidence of multidimensionally deprived individuals/households as well as the intensity of simultaneous deprivations. Firmly anchored in the AF methodology, the NDI also allows for decompositions along population and socio-economic dimensions^(8)^.

Our study applies the NDI to data from the Paraguayan Household Survey of Income and Expenditures 2011–2012, the most recent nationally representative survey that provides information on household food consumption. To the extent that household-level dietary diversity has been found to be strongly associated with household per capita income^(11)^, the study also examines the relationship between nutritional deprivation and household economic status (that is, the extent to which poorer households are at greater risk of nutritional deprivation than richer households), while controlling for a number of potentially confounding factors.

## Methods

The following section reviews the methodology underlying the construction of the NDI. As mentioned in the introduction, the methodology adjusts the AF technique for the use of household-level data by allowing us to consider minimum food group requirements for each member of the household based on their age, sex and other factors. The main components of the NDI include households' consumption matrix (*X*), the minimum consumption requirement matrix (*S_n_*) and the corresponding households' cut-off matrix (*Z*).

### Nutritional Deprivation Index

Let us first define an (*N* × *D*)-dimensional households' food groups consumption matrix:
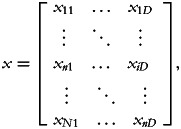
where the generic element *x_ij_* indicates the amount of food group *d* consumed by the *n*th household. That is, each row in *x* represents the amounts of food groups 1,…, *D* consumed by the *n*th household.

Next, for each household *n* = 1,…, *N* let us define an (*h_n_* × *d*)-dimensional matrix of household's minimum consumption requirements for the *h_n_* members of the *n*th household:
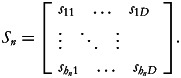
In this case, each row in *S_n_* corresponds to a distinct member of the *n*th household whose minimum consumption requirements are based on her/his characteristics (age, sex and/or other factors). Note that the dimension *h_n_* will vary from one household to another based on the number of individuals in the household.

The (*N* × *D*) matrix of households' cut-offs *Z* can then be obtained by placing at each row *n* of *Z* the corresponding row sum of the sub-matrix *S_n_*:
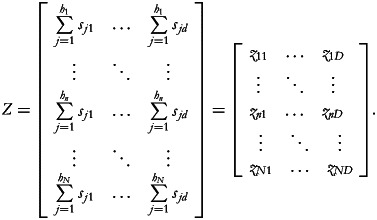
We can then compute the deprivation matrix *B* in which the generic element *b*_*nd*_ = 1 if *x*_*nd*_ < *z*_*nd*_ and *b*_*nd*_ = 0 otherwise.

Finally, given the vector of weights *w* = (*w*_1_, …, *w*_*D*_) (used to define the importance of each food group for a diverse diet), we can calculate the deprivation score for each household as:
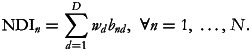
The values of the NDI, which fall within the range of 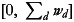, are higher the higher the number of food group deprivations. Note that if each food group is assigned an equal weight (*w*_*d*_ = 1 if normalised), the values of the NDI will fall within the range of [0, *D*].

Applying the second cut-off (corresponding to a minimum number of deprivations required to be considered malnourished), we obtain a binary version of the NDI, also referred to as the censored deprivation index:
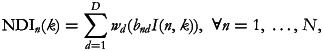
where 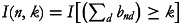 is an indicator function that assumes the value of 1 if the household *n* is deprived in at least *k* food groups and 0 otherwise.

Finally, we can construct the population-level measures. The intensity of deprivation can be obtained as:
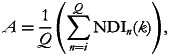
where 
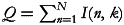
, whereas the incidence (headcount ratio) of households deprived in multiple dimensions as 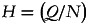. The adjusted headcount ratio is then obtained as:
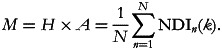


### National Income and Expenditure Survey 2011–2012

The data used in the present study were obtained from the National Income and Expenditure Survey of 2011–2012 (*Encuesta de Ingresos y Gastos* (EIG) 2011–2012). This was a nationally and sub-nationally representative national household survey conducted by the General Directorate of Statistics, Surveys and Censuses (DGEEC) between August 2011 and July 2012.

The survey collected demographic, socio-economic and expenditure data from a sample of 5417 households, of which 3446 (63 %) were urban and 1971 (37 %) were rural. These households contained a total of 21 130 individuals, implying an average size of a household of 3⋅9 members (one out of four households had six members or more). The survey used a two-stage stratified household design.

### Households’ consumption matrices

The use of the NDI requires the construction of the minimum consumption requirement matrix (*S_n_*), households' cut-off matrix (*Z*) and household consumption matrix (*X*).

We followed the healthy US-style eating pattern as a basis for the construction of households’ minimum consumption requirements matrix (*S_n_*)^(12)^. This pattern identifies recommended amounts of foods, in nutrient-dense forms, that an individual should consume from six major food groups (fruits, vegetables, grains, dairy products, protein foods and oils) and their sub-groups in order to meet nutrient and dietary guidelines standards, and also considers a limit on the maximum amount of energy available for other uses, such as added sugars, solid fats, added refined starches, or alcohol. The pattern considers twelve energy levels (from 1000 to 3200 kcal/d (4180 to 13 390 kJ/d)) to meet the needs of an individual across the lifespan.

We used the energy needs estimates provided by the Institute of Medicine to determine the energy level for each member of the household conditional on her age, sex and the level of physical activity^(13)^ (we restricted our analysis to the sedentary level of physical activity). These estimates are based on the estimated energy requirements equations, using reference heights and reference weights for each age–sex group. In other words, the household-specific minimum consumption requirement matrix is determined by vectors of age-, sex- and activity-specific recommended amounts for each household member. Applying the same procedure to all households then yields matrices *S*_1_,*…*, *S_N_* of households' minimum consumption requirements.

The households' cut-off matrix can be calculated as described in the previous section, with each row consisting of *D* sums of the minimum consumption requirements across all household members, where *D* represents the number of food groups. Again, recall that we do not consider individual-level cut-offs because the survey only provides consumption data at the aggregated, household-level (see the previous section).

Construction of the household's consumption matrix (*X*) requires the knowledge of the actual household consumption. Although our data do not provide the actual amounts of foods consumed by the household, they provide a detailed information about the quantities of (as well as the corresponding expenditures on) over 900 unique food items purchased or otherwise acquired by the households over the previous 7 d. For the purposes of our analysis, we only considered food items that the household either purchased or self-produced. Due to significant use of non-standard acquisition units, we did not consider food items that the household received from another household, from a social protection or nutrition programme, or as a gift from church or a non-profit institution, or that either member of the household took from a business. Of the total number of food items acquired by the households over the last 7 d, 90⋅1 % were purchased or self-produced; food items that either member of the household took from a business accounted for 5⋅36 %, food items that the household received from another household accounted for 3⋅82 %, and food items that the household received from a social protection or nutrition programme, or as a gift from church or a non-profit institution, accounted for the remaining <1 %.

Our analysis also excludes alcoholic and non-alcoholic drinks, sweets, spices, condiments and foods consumed away from home. Most foods consumed away from home include dishes whose serving sizes and ingredients are not standardised and thus would require making assumptions about both absolute and relative amounts of their individual food components. However, as these foods represent but a small fraction of households' expenditures in the data (evidencing the fact that eating out is far less common in Paraguay than in developed countries), we have no reason to believe that excluding them significantly impacts the results of the present study.

Using the data, we first classified food items into six food groups, including fruits, vegetables, grains, protein foods, dairy products, and oils^(8)^. Specifically, the fruits group was constructed by including all the fruit varieties, including fresh, frozen, canned and dried fruits and fruit juices (for example, bananas, grapes, raisins, oranges and orange juice); the vegetables group was constructed by including all the vegetable varieties in fresh, frozen, or canned form; and the proteins group was constructed by including all fish/seafood, meat, poultry, eggs, soya and soya products, nuts and seeds.

Next, we further split vegetables, grains and protein foods groups into food sub-groups. In particular, the vegetables group was classified into five sub-groups, including dark-green vegetables (for example, broccoli, collard greens, kale, spinach), red and orange vegetables (for example, carrots, pumpkin, red peppers, sweet potato, tomatoes), legumes (for example, black beans, garbanzos, green soyabeans, kidney beans, lentils, pinto beans, white beans), starchy vegetables (for example, cassava, green lima beans, green peas, plantains, potatoes) and other vegetables (for example, common lettuce, onion, cucumber, cabbage, celery, mushrooms, green peppers); the grains group was classified into two sub-groups, including whole grains and refined grains; and the protein foods group was classified into three sub-groups, including meats, eggs, soya and soya products, nuts and seeds. Meat and eggs represented by the far the most important constituents of the protein foods group, both in terms of volumes purchased and the relative expenditures. Soya-based products represented a small part of the group (in relative terms) as their consumption remains limited in Paraguay. Given that fruits, dairy products and oils groups remained a single item as before, the final classification included a total of thirteen groups or sub-groups.

For the purposes of our analysis, we converted each food item to its cup- (in case of fruits and vegetables) or ounce- (in case of protein foods) equivalents^(12)^. For fruits and vegetables, a cup-equivalent corresponds to one cup of vegetable or fruit, one cup of vegetable or fruit juice, two cups of leafy salad greens and 0⋅5 cup of dried fruit or vegetable. For protein foods, 1 ounce-equivalent corresponds to approximately 1 ounce of lean meat, poultry, or fish/seafood, one egg, one tablespoon of peanut butter and 0⋅5 ounce of nuts or seeds. We applied the Food Patterns Equivalents Ingredients Database (FPID) cup-equivalent weights and, where appropriate, the FPID in combination with ARS Food Intakes Converted to Retail Commodities Database (FICRCD) conversion factors to estimate the amount of raw fruits and vegetables to be purchased in order to obtain one cup-equivalent of raw (edible) portion of each food item^(14,15)^. The weight/volume of the particular food item can vary significantly depending on whether it is consumed raw or prepared (boiled/cooked). Therefore, for each food item traditionally consumed in a cooked state (such as pumpkin, lentils, meats), we converted the raw amounts to cooked amounts using a yield factor^(16,17)^. We used Internet resources to determine the yield factors for the food items that were not available in the manuals. For the meats, we fixed the yield factor at 0⋅8.

Finally, the household's consumption matrix (*X*) was obtained by adding the household's apparent consumption of food items across each food group (sub-group).

### Statistical analyses

Estimates of the NDI (and the related measures) were calculated according to households’ income quintiles and area of residence (rural or urban). Differences among groups were analysed using the χ^2^ test. Where relevant, linear trends across income quintiles and areas were assessed. In all analyses, the data were weighted using the expansion factors provided in the EIG datasets. The analysis was performed in R^TM^ statistical software, version 3.4.3, using the package ‘survey’^(18)^.

The EIG dataset contains detailed information on household income and expenditures. In this study, the monthly per capita household income was used to stratify households into five income quintiles (Q1–Q5). The corresponding income quintile thresholds were as follows: Q1: Paraguyan guarani (Gs.) 0 to 353 992; Q2: Gs. 354 262 to 610 327; Q3: Gs. 610 784 to 930 532; Q4: Gs. 930 913 to 1⋅514⋅103; and Q5: Gs. 1 515 036 and more.

The effects of household income on nutritional deprivation were estimated after statistically controlling for the effects of a number of potentially confounding factors. The factors include household size (0–4, 5–8 and 9 members or more), language spoken by the household head (Spanish, Guaraní, bilingual, or other), education level of female and male household head (no education, primary school education or less, middle school education or less and secondary school education or higher), household's area of residence (rural/urban) and the department (country's basic administrative division). The definition of each variable is provided in the Appendix (Table A1).

The effects of household economic status and other factors on nutritional deprivation were estimated using a multivariate logistic regression procedure. A number of alternative models were estimated to assess the relative significance of various confounding factors included in the analysis, as well as the robustness of the results. Results of multivariate analyses are presented as OR with 95 % CI.

A final note concerns the construction of the dependent variable. For the estimation purposes, the NDI was transformed into a binary variable. Given this transformation is dependent on the value of *k* (minimum number of food group deprivations), the logistic regression analysis was performed varying the parameter from *k* = 4 to *k* = 6. The results of the analysis for *k* = 5 and *k* = 6 were qualitatively similar to those for *k* = 4 presented in the text.

## Results

Table 1 provides basic descriptive statistics for the data sample used in the analysis. The table highlights important differences between rural and urban data samples in terms of the income distribution, household size and education of and languages spoken by household heads. Understanding these differences can serve as a useful reference both for the analysis of rural–urban differences in the incidence and intensity of nutritional deprivation and the association of nutritional deprivation with various household characteristics discussed further in the study. In particular, the table shows that a relatively larger share of households in the rural sample is poor (55 % fall in the bottom two income quintiles), while a relatively larger share of households in the urban sample is rich (58 % fall in the top two income quintiles). In terms of household size, rural households are on average larger than urban households; this is mainly due to a larger share of larger households in rural areas compared with urban areas (38⋅5 % of rural households have five or more members compared with 30 % of urban households). In terms of the language(s) spoken by the heads of household, only one in four (24 %) of those in rural households speak Spanish compared with about two in three (67 %) in urban households. Guaraní as the sole language is spoken by two in three (69 %) heads of household in rural areas, but only one in three (31 %) heads of household in urban areas. Finally, rural households exhibit a significantly lower educational attainment for their household heads, with about twice as many of those in rural households than those in urban households having no or primary education, and between two to three times as many households heads in urban areas than in rural areas having middle or higher education.

**Table 1.** Descriptive statistics*

	Global	Rural	Urban
Households			
*n*	5417	1971	3446
%	–	63·6	36·4
Economic status			
1st quintile (poorest)	16·8	31·1	8·6
2nd quintile	18·4	23·6	15·4
3rd quintile	17·9	17·8	18·0
4th quintile	20·6	14·6	24·0
5th quintile (richest)	26·4	12·9	34·1
Household size			
Average size (*n*)	3·9	4·1	3·7
0–4 persons	67·0	61·5	70·1
5–8 persons	29·9	34·5	27·4
>8 persons	3·1	4·0	2·6
Language			
Spanish	20·9	6·1	29·4
Guaraní	45·2	69·4	31·3
Bilingual	30·5	18·3	37·5
Other	3·4	6·2	1·7
Male household education level			
None	4·9	8·4	2·6
Primary	46·5	65·3	34·6
Middle	32·9	21·8	40·0
Secondary or higher	15·7	4·5	22·8
Female household education level			
None	7·3	11·6	4·9
Primary	48·7	66·9	38·3
Middle	27·4	16·2	33·7
Secondary or higher	16·6	5·3	23·1

* Descriptive statistics for the full sample and rural/urban households. All numbers are expressed in percentage points (%) except for the total number of households and the average size of the household; the latter shows the average number of persons living in the household. See Table A1 in the Appendix for variable definitions. Source: authors’ calculations.

### Food group analysis

As a starting point, we analyse simple (population-level) headcount ratios; these ratios do not communicate the incidence of multidimensionally deprived, but only the incidence of deprivation in each food group. At the food-group level, the simple headcount ratios show that most Paraguayan households were deprived in dairy products (82 %), followed by fruits (69 %), proteins (56 %) and vegetables (53 %); only 25 % of households were deprived in cereals (Table 2). More urban than rural households were deprived in vegetables (62 *v.* 38 %; *P* < 0·001), whereas more rural than urban households were deprived in grains (29 *v.* 23 %; *P* < 0·001) and proteins (60 *v.* 54 %; *P* < 0·001).

**Table 2.** Simple headcount ratios for basic food groups by income quintiles (Q)†

Food group	Area				Income quintile						
	Global	Rural	Urban	DF	Q1	Q2	Q3	Q4	Q5	DF	TR
Vegetables	0·53	0·38	0·62	***	0·40	0·48	0·54	0·58	0·61	***	***
Greens	0·86	0·89	0·84	***	0·91	0·88	0·85	0·84	0·83	***	***
Red and orange	0·87	0·91	0·85	***	0·95	0·91	0·89	0·86	0·80	***	***
Legumes	0·82	0·74	0·86	***	0·74	0·78	0·83	0·85	0·87	***	***
Starchy	0·46	0·27	0·57	***	0·26	0·36	0·44	0·51	0·64	***	***
Other	0·60	0·67	0·56	***	0·76	0·69	0·61	0·54	0·47	***	***
Fruits	0·69	0·69	0·68		0·76	0·75	0·72	0·68	0·59	***	***
Grains	0·25	0·29	0·23	***	0·30	0·25	0·21	0·23	0·28	***	***
Whole	0·95	0·99	0·93	***	0·99	0·99	0·97	0·94	0·89	***	***
Refined	0·10	0·12	0·09	**	0·11	0·08	0·06	0·07	0·15	***	***
Dairy products	0·82	0·82	0·82		0·93	0·88	0·82	0·80	0·72	***	***
Proteins	0·56	0·60	0·54	***	0·72	0·60	0·54	0·52	0·48	***	***
Seafood	0·94	0·94	0·94	***	0·96	0·95	0·94	0·94	0·91	***	***
Meats	0·38	0·43	0·35	***	0·57	0·38	0·33	0·33	0·32	***	***
Nuts	0·94	0·91	0·96	***	0·91	0·94	0·95	0·96	0·95	***	***
Oils	1·00	1·00	1·00		1·00	1·00	1·00	1·00	1·00		
Observations (*n*)	5417	1971	3446		908	995	971	1414	1429		

** *P* *<* 0*.*01, *** *P* *<* 0*.*001.

† Simple headcount ratios for basic food groups and food subgroups for the whole sample, and by rural/urban areas and household income. DF (difference test): *H*_0_: the deprivation proportion is the same in each group. *H_a_*: at least one deprivation proportion is different from the others. TR (linear trend test): *H*_0_: no linear trend in the deprivation proportions across groups. *H_a_*: linear trend in the deprivation proportion across groups. Source: authors’ calculations.

The analysis of population-level headcount ratios shows important food sub-group variation within the corresponding food groups. For example, whereas 53 % of Paraguayan households were found to be deprived in vegetables, the food sub-group analysis shows that only 46 % of households were deprived in starches, but as many as 86 % of households were deprived in green vegetables and 87 % of households in red and orange vegetables. Similarly, notable variations can be observed within grains and proteins sub-groups.

The rural–urban differences also remain evident at the food sub-group level, with more rural than urban households found to be deprived in green vegetables (89 *v.* 84 %; *P* < 0·001), red and orange vegetables (91 *v.* 85 %; *P* < 0·001), whole grains (99 *v.* 93 %; *P* < 0·001) and meats (43 *v.* 35 %; *P* < 0·001), among others; conversely, more urban than rural households were found to be deprived in legumes (86 *v.* 74 %; *P* < 0·001), starchy vegetables (57 *v.* 27 %; *P* < 0·001) and nuts (96 *v.* 91 %; *P* < 0·001), among others.

The simple headcount ratios varied significantly with household economic status. In particular, for fruits, dairy products and proteins, the simple incidence of deprivation declined monotonically with increasing income, while for vegetables, the simple incidence of deprivation increased monotonically with increasing income (Table 2). For example, 72 % of Q1, 54 % of Q3 and 48 % of Q5 households were deprived in proteins. Similar findings were obtained when the analysis was carried out separately for rural and urban households, although no relationship was found between the incidence of deprivation and the level of income in vegetables group in urban areas (Appendix: Table A2).

In general, the simple headcount ratios for the food sub-groups followed the same monotonic behaviour as that observed for basic food groups. In some instances, however, this behaviour was contrary to the latter: in particular, for green vegetables and red and orange vegetables, the simple headcount ratio declined monotonically with increase in income, and for nuts and seeds group, the simple headcount ratio increased monotonically with increase in income. Similar findings were obtained when the analysis was carried out separately for rural and urban households (Appendix: Table A2).

Joint analysis of household's economic status and its area of residence shows that differences in simple incidence of deprivation between rural and urban households were in general more significant in lower income quintiles (Table 3). In particular, whereas the lowest-income households (Q1−Q2) showed significant differences in four out of six food groups (*P* < 0·05), the middle-income households (Q3) showed differences in three food groups, and the highest-income households (Q4−Q5) in only one to two groups.

**Table 3.** Simple headcount ratios for basic food groups and food sub-groups by income quintiles (Q) and rural/urban areas†

Food group	Q1		Q2		Q3		Q4		Q5	
	Rural	Urban	Rural Urban		Rural	Urban	Rural	Urban	Rural	Urban
Vegetables	0·32	0·56***	0·35	0·60***	0·37	0·63***	0·47	0·63***	0·51	0·63***
Greens	0·92	0·89	0·88	0·89	0·86	0·84	0·87	0·82	0·89	0·82**
Red and orange	0·96	0·92*	0·91	0·91	0·89	0·89	0·89	0·84	0·84	0·80
Legumes	0·70	0·82***	0·71	0·84***	0·75	0·87***	0·80	0·87**	0·80	0·88***
Starchy	0·18	0·41***	0·24	0·47***	0·27	0·53***	0·35	0·57***	0·45	0·68***
Other	0·81	0·66***	0·71	0·67	0·62	0·60	0·57	0·53	0·49	0·47
Fruits	0·73	0·81**	0·71	0·78*	0·69	0·74	0·65	0·69	0·62	0·58
Grains	0·34	0·22**	0·30	0·21**	0·21	0·21	0·27	0·22	0·31	0·27
Whole	0·99	0·98	1·00	0·97**	0·99	0·96**	0·98	0·93**	0·95	0·88**
Refined	0·13	0·06**	0·12	0·05***	0·06	0·06	0·10	0·07	0·17	0·15
Dairy products	0·92	0·94	0·84	0·92***	0·75	0·87***	0·75	0·82**	0·70	0·72
Proteins	0·75	0·65**	0·60	0·59	0·49	0·57*	0·52	0·53	0·46	0·48
Seafood	0·97	0·96	0·94	0·96	0·92	0·95	0·93	0·95	0·91	0·91
Meats	0·61	0·51*	0·43	0·35*	0·29	0·35	0·35	0·33	0·32	0·32
Nuts	0·88	0·97***	0·91	0·97***	0·92	0·97**	0·95	0·96	0·92	0·96**
Oils	1·00	1·00	1·00	1·00	1·00	1·00	1·00	1·00	1·00	1·00
Observations (*n*)	613	295	465	530	350	621	288	826	255	1174

* *P* *<* 0*.*05, ** *P* *<* 0*.*01, *** *P* *<* 0*.*001.

† Simple headcount ratios for basic food groups and food subgroups by income quintiles and rural/urban areas. Asterisks denote the results of the difference test between rural and urban groups (*H*_0_: the deprivation proportion is the same in each group. *H_a_*: the deprivation proportions are different.) Source: authors' calculations.

Table 4 reports incidence of deprivation (headcount ratio) (*H*), intensity of deprivation (*A*) and adjusted headcount ratio (*M*) for values of *k* ranging from 0 to 6. Recall that the value of *k* represents the minimum number of food group deprivations necessary to be considered deprived. Thus, the lower the value of *k*, the higher the incidence of nutritionally deprived households; that is, the headcount ratio *H* tends to 1, or 100 %.

**Table 4.** Incidence of deprivation, intensity of deprivation and adjusted headcount ratio for the whole sample and by rural/urban area*

	Global			Rural			Urban		
*k*	*H*	*A*	*M*	*H*	*A*	*M*	*H*	*A*	*M*
0	1·00	0·75	0·75	1·00	0·75	0·75	1·00	0·75	0·75
1	1·00	0·75	0·75	1·00	0·75	0·75	1·00	0·75	0·75
2	0·98	0·76	0·74	0·98	0·76	0·74	0·98	0·76	0·75
3	0·90	0·78	0·70	0·88	0·78	0·69	0·90	0·78	0·70
4	0·67	0·82	0·55	0·66	0·82	0·54	0·64	0·82	0·53
5	0·31	0·87	0·27	0·32	0·87	0·28	0·29	0·87	0·25
6	0·03	1·00	0·03	0·03	1·00	0·03	0·02	1·00	0·02

* Incidence of deprivation (*H*), intensity of deprivation (*A*) and adjusted headcount ratio (*M*) conditional on the value of *k* (minimum number of group deprivations) for the whole sample (*n* 5417), rural area (*n* 1971) and urban area (*n* 3446). Source: authors’ calculations.

Note that while the analysis is based on thirteen groups and sub-groups, the results are presented for *k* varying from 0 to 6, the number of food groups. A brief explanation is in order. Recall that the calculation of the NDI requires the definition of the weights *w_d_* for each food group. In the analysis, we considered each food group to be as important as any other food group; therefore, each food group was assigned an equal weight (*w_d_* = 1 if normalised). Analogously, within a given food group, each food sub-group was considered to be as important as any other food sub-group, so that, for example, each vegetable food sub-group received an equal weight of 1/5 and the sum of their weights summed up to 1. This approach ensures that consumption of the starchy sub-group, for example, cannot in itself satisfy the entire vegetable food group requirement.

The results show that every Paraguayan household was nutritionally deprived in at least one food group (*k* = 1, *H* = 1). This is, in fact, the oil group, as seen in Table 2. In this case, the intensity of deprivation is 0·75, implying that the same households were on average nutritionally deprived in about 4·5 food groups. Similarly, just over three out of every five households (67 %) were inadequately nourished in at least four food groups. The corresponding intensity of deprivation was close to five food groups (4·92). There were no differences in the incidences of deprivation for different values of *k* between urban and rural households.

The incidence of deprivation decreased monotonically with the level of household income. Moving from the lowest (Q1) to the highest income quintile (Q2), the proportion of households that were inadequately nourished decreased from 99 to 95 % for *k* = 2, from 94 to 83 % for *k* = 3, from 74 to 60 % for *k* = 4 and from 34 to 30 % for *k* = 5 (Table 5). Similar results were observed when the analysis was carried out separately for rural and urban households (Appendix: Table A3).

**Table 5.** Incidence and intensity of deprivation and adjusted headcount ratio by income quintiles†

	Q1			Q2			Q3			Q4			Q5			
*k*	*H*	*A*	*M*	*H*	*A*	*M*	*H*	*A*	*M*	*H*	*A*	*M*	*H*	*A*	*M*	TR
0	1·00	0·79	0·79	1·00	0·77	0·77	1·00	0·75	0·75	1·00	0·74	0·74	1·00	0·71	0·71	
1	1·00	0·79	0·79	1·00	0·77	0·77	1·00	0·76	0·75	1·00	0·74	0·74	1·00	0·71	0·71	
2	0·99	0·80	0·79	0·99	0·78	0·77	0·99	0·76	0·75	0·97	0·75	0·73	0·95	0·73	0·69	***
3	0·94	0·81	0·76	0·93	0·79	0·74	0·90	0·78	0·70	0·87	0·78	0·68	0·83	0·76	0·63	***
4	0·74	0·83	0·62	0·73	0·82	0·59	0·67	0·81	0·55	0·65	0·82	0·54	0·60	0·81	0·48	***
5	0·34	0·88	0·30	0·29	0·88	0·25	0·30	0·87	0·26	0·30	0·87	0·26	0·30	0·87	0·26	**
6	0·02	1·00	0·02	0·02	1·00	0·02	0·01	1·00	0·01	0·03	1·00	0·03	0·04	1·00	0·04	

** *P* < 0·01, *** *P* < 0·001.

† Incidence of deprivation (*H*), intensity of deprivation (*A*) and adjusted headcount ratio (*M*) conditional on the value of *k* (minimum number of group deprivations) by income quintiles (Q1−Q5). Asterisks denote the results of the test for the presence of the linear trend (TR) in the incidence of deprivation across income quintiles. Number of observations: *n_Q1_* = 908, *n_Q2_* = 995, *n_Q3_* = 971, *n_Q4_* = 1,114 and *n_Q5_* = 1429. *Source*: authors’ calculations.

Table 6 provides analysis of the percentage contribution of each food sub-group to the incidence of deprivation for *k* = 4. We find that, apart from oils, dairy products and fruits contributed the most to the incidence of deprivation relative to other food groups. Separating the rural and urban households, the differences in percentage contributions are – with the exception of starch – generally marginal. However, an interesting pattern is found at different income quintiles, with legumes and starch (meats), for example, contributing more (less) to the incidence of deprivation the higher the household income.

**Table 6.** Percentage contribution of food sub-groups to incidence of deprivation (*k* = 4)*

		Area		Income quintile				
Food group	Global	Rural	Urban	Q1	Q2	Q3	Q4	Q5
Vegetables								
Greens	9·10	8·89	9·48	9·26	9·21	9·04	8·96	9·07
Red and orange	9·28	9·01	9·75	9·63	9·52	9·47	9·17	8·82
Legumes	8·68	9·14	7·89	7·54	8·10	8·79	9·09	9·49
Starchy	4·90	6·04	2·90	2·62	3·78	4·63	5·50	6·98
Other	6·36	5·89	7·19	7·70	7·17	6·49	5·80	5·20
Fruits								
Any	7·30	7·25	7·38	7·69	7·79	7·64	7·29	6·43
Grains								
Whole	10·07	9·81	10·52	10·06	10·27	10·34	10·10	9·70
Refined	1·04	0·94	1·23	1·12	0·84	0·61	0·80	1·64
Dairy products								
Any	8·70	8·70	8·71	9·41	9·19	8·78	8·60	7·89
Proteins								
Seafood	9·95	9·90	10·02	9·81	9·88	10·01	10·10	9·92
Meats	4·02	3·70	4·58	5·76	4·01	3·48	3·59	3·54
Nuts	10·02	10·20	9·70	9·24	9·83	10·09	10·27	10·43
Oils								
Any	10·59	10·56	10·66	10·16	10·41	10·63	10·71	10·43
Observations (*n*)	5417	1971	3446	908	995	971	1414	1429

* Percentage contribution of food subgroups to incidence of deprivation (*H*) for *k* = 4. Source: authors’ calculations.

### Effect of household income on nutritional deprivation

The unadjusted odds of nutritional deprivation are more than three times higher among the lowest income (Q1) households than among the highest income (Q5) households (OR 3·0; 95 % CI 2·3, 3·9) (Table 7, model 1). Similarly, Q2 households also face higher odds of nutritional deprivation than Q5 households, although the income effect is not as pronounced (OR 2·3; 95 % CI 1·8, 2·9). The middle-income quintile households (Q3 and Q4) face marginally higher risk of nutritional deprivation than Q5 households. The relationship remains qualitatively unchanged when controlling for household size, language spoken by the household head and the education level of female/male head of household (model 2), as well as for household's residence (rural/urban) (model 3) and the department (model 4). In other words, the poorest 40 % of households are about two to three times more likely to be nutritionally deprived than the richest 60 % of households (Q1 households: OR 3·1; 95 % CI 2·1, 4·5 and Q2 households: OR 2·3; 95 % CI 1·7, 3·2).

**Table 7.** Estimates of the effects of household income and other household characteristics on nutritional deprivation for *k* = 4 and thirteen food groups/sub-groups* (Odds ratios and 95 % confidence intervals)

	Model 1		Model 2		Model 3		Model 4	
Variable	OR	95 % CI	OR	95 % CI	OR	95 % CI	OR	95 % CI
Intercept	6·3	5·2, 7·6	8·8	3·0, 25·6	9·4	3·2, 27·6	12·3	3·8, 39·6
Economic status								
5th quintile (richest)†	–	–	–	–	–	–	–	–
4th quintile	1·3	1·0, 1·6	1·3	1·0, 1·7	1·3	1·0, 1·7	1·3	1·0, 1·8
3rd quintile	1·5	1·2, 1·8	1·2	0·9, 1·6	1·2	0·9, 1·7	1·3	0·9, 1·7
2nd quintile	2·3	1·8, 2·9	2·0	1·5, 2·8	2·2	1·6, 3·0	2·3	1·7, 3·2
1st quintile (poorest)	3·0	2·3, 3·9	2·3	1·6, 3·4	2·7	1·9, 4·0	3·1	2·1, 4·5
Household size								
0–4 persons†			–	–	–	–	–	–
5–8 persons			1·6	1·3, 1·9	1·6	1·3, 1·9	1·5	1·2, 1·9
>8 persons			3·5	1·9, 6·7	3·5	1·8, 6·6	3·4	1·8, 6·6
Language								
Spanish†			–	–	–	–	–	–
Guraní			0·8	0·6, 1·1	0·9	0·6, 1·2	0·9	0·7, 1·3
Bilingual			0·7	0·5, 1·0	0·8	0·6, 1·0	0·8	0·6, 1·0
Others			1·2	0·7, 2·1	1·5	0·8, 2·8	1·4	0·7, 2·7
Male household education level								
None†			–	–	–	–	–	–
Primary			1·2	0·8, 1·9	1·2	0·8, 1·9	1·2	0·8, 1·9
Middle			1·1	0·7, 1·8	1·0	0·6, 1·7	1·0	0·7, 1·7
Secondary or higher			1·1	0·6, 1·9	1·0	0·6, 1·8	1·0	0·6, 1·7
Female household education level								
None†			–	–	–	–	–	–
Primary			0·5	0·3, 0·8	0·5	0·3, 0·8	0·5	0·3, 0·8
Middle			0·4	0·3, 0·8	0·4	0·3, 0·7	0·4	0·2, 0·7
Secondary or higher			0·4	0·2, 0·6	0·4	0·2, 0·6	0·4	0·2, 0·6
Area								
Urban†					–	–	–	–
Rural					0·6	0·5, 0·8	0·7	0·5, 0·9
Department								
Asunción†							–	–
San Pedro							0·5	0·3, 0·8
Caaguazú							0·5	0·3, 0·8
Itapúa							0·6	0·4, 1·0
Alto Paraná							0·9	0·6, 1·4
Central							0·9	0·6, 1·2
Other departments							0·8	0·6, 1·2

* Pooled OR estimates and the associated 95 % CI from the multivariate logistic regression of the Nutritional Deprivation Index on household income and other household characteristics; each model was run with 5417 observations. See Table A1 in the Appendix for variable definitions. Source: authors’ calculations.

† Reference group.

### Effects of other factors and confounders

Among the control variables, household size has the strongest effect on the risk of nutritional deprivation, and this effect is independent of household income and other household characteristics (Table 7, models 2–4). With household income and other factors controlled, households headed by Guaraní speakers (both monolingual and bilingual) are significantly less likely to be nutritionally deprived than households whose heads speak a language other than Spanish and/or Guaraní. Also, the adjusted prevalence of nutritional deprivation is significantly lower among households whose female head has some education than among households whose female head is uneducated, although this effect is relatively small. In contrast, the education level of the male head of household has no apparent effect on household's nutritional deprivation.

Finally, the adjusted prevalence of nutritional deprivation is significantly lower among households from relatively less populated departments (Caaguazú: OR 0·5, 95 % CI 0·3, 0·8; Itapúa: OR 0·6, 95 % CI 0·4, 1·0; San Pedro: OR 0·5, 95 % CI 0·3, 0·8), compared with households from Paraguay's most populated departments (Asunción and the department of Central). Households residing in rural areas are marginally less likely to be nutritionally deprived than urban households (models 3–4).

We also estimated the above regressions separately for urban and rural areas. The results are similar to those obtained in pooled analysis (Appendix: Table A4). In particular, household economic status continues to have a similar effect on nutritional deprivation as that found in pooled regressions, although this effect is somewhat less pronounced in rural areas. Household size continues to have the strongest effect on the risk of nutritional deprivation; in contrast to household's economic status, this effect is the strongest in rural areas.

## Discussion

Lack of dietary diversity, particularly severe among poor populations, has become increasingly relevant in light of the recent shifts in global dietary and activity patterns^(19)^. More diverse diets also tend to be associated with lower rates of overweight and obesity – nutritional problems of rising magnitude in many parts of the world^(20)^. Increasing dietary diversity therefore constitutes an important strategy to improve nutrition and health.

The present study takes a novel approach to measuring access to diverse diets by applying the recently proposed NDI to nationally representative data of Paraguayan households. Results of this study show that Paraguayan households were significantly deprived across most food groups. The study also finds that, with the exception of vegetables, the level of nutritional deprivation decreased monotonically with income. Further results from the logistic regressions confirm that poorer households were at a greater risk of being nutritionally deprived than higher-income households. These findings contribute to a growing literature analysing the association between household economic status and dietary diversity^(11)^. More importantly, the findings suggest that strategies are needed to improve households' access to diverse diets, especially among their lower- and middle-income segments.

Building on the AF methodology traditionally applied in multidimensional poverty measurement, the NDI framework applied in this study overcomes the main weaknesses of conventional dietary diversity indices in that it allows us to measure both the incidence and the average deprivation share of the inadequately nourished^(7–9)^. Moreover, the NDI framework also incorporates individual-specific thresholds, allowing the consumption to vary by age, sex and other factors. As noted by Oldiges^(9)^, the NDI framework has many attractive properties. From the policy perspective, perhaps the most useful property of this framework is its ability to separately analyse specific regions or population segments. For example, the NDI framework allows us to isolate population groups or regions that are not adequately nourished, while also identifying the specific food groups absent in the diet. This makes the framework well suited for targeting purposes. Furthermore, the framework may be used as a basis for the design of policies aimed at improving food security and nutrition outcomes. To the extent that the NDI allows us to identify the (contribution of) specific food groups to overall incidence of nutritional deprivation within specific demographic and socio-economic strata, policies aimed at promoting healthier diet patterns may focus only on particular food (sub-) groups or population.

The main shortcomings of the present study are related mainly to the dataset used in the analysis. The use of apparent consumption data (as purchased or otherwise obtained by the household over the recall period) as a proxy for actual food consumption entails making at least two assumptions about household food consumption profile. These include (1) that the food acquired by the households is (actually) consumed and (2) that all the food acquired during the recall period is consumed during the same period^(21)^. The latter assumption also implies that food acquired prior to the recall period is consumed prior to the start of the recall period.

The enquiry about the food items acquired by members of the household over a specific period of time is further subject to reporting error due to poor recollection over long periods of time and, at a more general level, due to various non-observation (non-sampling) measurement errors^(8,22)^. These measurement errors can be further compounded when foods consumed away from home, which generally constitute a non-trivial part of households' apparent consumption, are included in the analysis, as such inclusion involves making assumptions about both absolute and relative amounts of the specific ingredients entering their preparation. Nevertheless, our study avoids this particular challenge; foods consumed away from home represent but a small fraction of food expenditures of the Paraguayan households and as their inclusion would have immaterial impact on the results, they are excluded from the analysis. Finally, the use of household-level consumption data also does not allow us to consider intra-household inequalities in food consumption, nor to precisely capture the person-specific differences^(23,24)^.

## Appendix

**Table A1.** Variable definitions

Variable name	Definition
Economic status	Quintiles of monthly per capita household income
Household size	Indicator variable for number of members in a household: 0–4, 5–8 and >8 members of household
Maternal education	Indicator variable for maximum years of schooling achieved by any female adult (aged 16+ years) household member: no education (reference), primary education or less, middle education or less, secondary education and more
Paternal education	Indicator variable for maximum years of schooling achieved by any male adult (aged 16+ years) household member: no education (reference), primary education or less, secondary education or more
Head Spanish (reference)	Indicator variable = 1 if head of the household speaks monolingual Spanish
Head Guaraní	Indicator variable = 1 if head of the household speaks monolingual Guaraní
Head bilingual	Indicator variable = 1 if head of the household speaks bilingual Spanish and Guaraní
Head other language	Indicator variable = 1 if head of the household speaks a language other than Spanish or Guaraní
Area	Indicator variable: urban area (reference) and rural area
Departments	Indicator variables for departments of Asuncion (reference), San Pedro, Caaguazix, Itapiia, Alto Parana, Central and Rest (a representative grouping of the remaining departments)

**Table A2.** Simple headcount ratios for basic food groups by household area of residence and economic status†

Food group	All	Q1	Q2	Q3	Q4	Q5	DF	TR
Rural areas								
Vegetables	0·38	0·32	0·35	0·37	0·47	0·51	***	***
Greens	0·89	0·92	0·88	0·86	0·87	0·89	*	*
Red and orange	0·91	0·96	0·92	0·89	0·89	0·85	***	***
Legumes	0·74	0·70	0·71	0·75	0·80	0·80	**	***
Starchy	0·27	0·18	0·24	0·27	0·35	0·45	***	***
Other	0·67	0·80	0·71	0·62	0·57	0·49	***	***
Fruits	0·69	0·73	0·71	0·69	0·65	0·62	**	***
Grains	0·29	0·34	0·30	0·21	0·27	0·31	***	*
Whole	0·99	0·99	1·00	0·99	0·98	0·95	***	***
Refined	0·12	0·13	0·12	0·06	0·10	0·17	***	
Dairy products	0·82	0·92	0·84	0·75	0·75	0·70	***	***
Proteins	0·60	0·75	0·60	0·49	0·52	0·46	***	***
Seafood	0·94	0·97	0·94	0·92	0·93	0·91	**	***
Meats	0·43	0·60	0·42	0·29	0·35	0·32	***	***
Nuts	0·91	0·88	0·91	0·92	0·95	0·92	*	**
Oils	1·00	1·00	1·00	1·00	1·00	1·00		
Observations (*n*)	1971	613	465	350	288	255		
Urban areas								
Vegetables	0·62	0·56	0·60	0·63	0·63	0·63		*
Greens	0·84	0·89	0·89	0·84	0·82	0·82	***	***
Red and orange	0·85	0·91	0·91	0·89	0·85	0·8	***	***
Legumes	0·86	0·82	0·84	0·87	0·87	0·88	*	**
Starchy	0·57	0·41	0·47	0·53	0·57	0·68	***	***
Other	0·56	0·66	0·67	0·60	0·53	0·47	***	***
Fruits	0·68	0·81	0·78	0·74	0·69	0·58	***	***
Grains	0·23	0·22	0·21	0·21	0·22	0·27	**	**
Whole	0·93	0·98	0·97	0·96	0·93	0·88	***	***
Refined	0·09	0·06	0·05	0·06	0·07	0·15	***	***
Dairy products	0·82	0·94	0·92	0·87	0·82	0·72	***	***
Proteins	0·54	0·65	0·59	0·57	0·53	0·48	***	***
Seafood	0·94	0·96	0·96	0·95	0·95	0·91	***	***
Meats	0·35	0·51	0·35	0·35	0·33	0·32	***	***
Nuts	0·96	0·97	0·97	0·97	0·96	0·96		
Oils	1·00	1·00	1·00	1·00	1·00	1·00		
Observations (*n*)	3446	295	530	621	826	1174		

** P <* 0·05, ** *P <* 0·01, *** *P* < 0·001.

† Simple headcount ratios for basic food groups and food sub-groups by household area of residence and income quintile (Q1–Q5). DF (difference test): *H_0_*: the deprivation proportion is the same in each group. *H_a_*: at least one deprivation proportion is different from the others. TR (linear trend test): *Hq*: no linear trend in the deprivation proportion across groups. *H_a_*: linear trend in the deprivation proportion across groups. Source: authors’ calculations.

**Table A3.** Incidence of deprivation, intensity of deprivation and adjusted headcount ratio by household area of residence and economic status†

	Q1			Q2			Q3			Q4			Q5			
*k*	*H*	*A*	*M*	*H*	*A*	*M*	*H*	*A*	*M*	*H*	*A*	*M*	*H*	*A*	*M*	TR
Rural area																
0	1·00	0·79	0·79	1·00	0·76	0·76	1·00	0·73	0·73	1·00	0·73	0·73	1·00	0·71	0·71	
1	1·00	0·79	0·79	1·00	0·76	0·76	1·00	0·73	0·72	1·00	0·73	0·73	1·00	0·71	0·71	
2	0·99	0·79	0·79	0·98	0·76	0·75	0·97	0·73	0·71	0·97	0·74	0·71	0·96	0·73	0·70	
3	0·96	0·80	0·77	0·91	0·78	0·70	0·87	0·76	0·65	0·86	0·76	0·66	0·84	0·75	0·63	**
4	0·74	0·83	0·61	0·68	0·81	0·55	0·59	0·80	0·47	0·59	0·81	0·48	0·56	0·81	0·46	***
5	0·33	0·89	0·30	0·26	0·88	0·23	0·22	0·86	0·19	0·30	0·87	0·26	0·28	0·87	0·25	**
6	0·02	1·00	0·02	0·02	1·00	0·02	0·01	1·00	0·01	0·03	1·00	0·03	0·02	1·00	0·02	
Urban area																
0	1·00	0·80	0·80	1·00	0·79	0·79	1·00	0·77	0·77	1·00	0·75	0·75	1·00	0·71	0·71	
1	1·00	0·80	0·80	1·00	0·79	0·79	1·00	0·77	0·77	1·00	0·75	0·75	1·00	0·71	0·71	
2	0·99	0·81	0·80	0·98	0·79	0·78	0·99	0·77	0·76	0·99	0·75	0·74	0·96	0·72	0·70	***
3	0·94	0·82	0·77	0·93	0·80	0·75	0·92	0·79	0·72	0·89	0·78	0·69	0·84	0·76	0·63	***
4	0·75	0·85	0·63	0·73	0·83	0·61	0·68	0·83	0·56	0·65	0·82	0·54	0·61	0·81	0·49	***
5	0·38	0·89	0·34	0·34	0·88	0·30	0·33	0·88	0·30	0·28	0·88	0·25	0·29	0·88	0·25	**
6	0·01	1·00	0·01	0·02	1·00	0·02	0·02	1·00	0·02	0·02	1·00	0·02	0·05	1·00	0·05	*

* *P* < 0·05, ** *P* < 0·01, *** *P* < 0·001.

† Incidence of deprivation (*H*), intensity of deprivation (*A*) and adjusted headcount ratio (*M*) conditional on the value of *k* (minimum number of group deprivations) by rural/urban area and income quintiles (Q1–Q5). Asterisks denote the results of the test for the presence of the linear trend in the incidence of deprivation across income quintiles. Number of observations for rural areas: *n*_Q1_ = 613, *n*_Q2_ = 465, *n*_Q3_ = 350, *n*_Q4_ = 288 and *n*_Q5_ = 255. Number of observations for urban areas: *n*_Q1_ = 295, *n*_Q2_ = 530, *n*_Q3_ = 621, *n*_Q4_ = 826 and *n*_Q5_ = 1174. Source: authors’ calculations.

**Table A4.** Robustness analysis – urban and rural regressions: OR estimates of the effects of household income and other household characteristics on nutritional deprivation for *k* = 4 and thirteen food groups/sub-groups*

	Urban				Rural			
	Model 1		Model 2		Model 1		Model 2	
Variable	OR	95 % CI	OR	95 % CI	OR	95 % CI	OR	95 % CI
Intercept	3·2	2·6, 3·8	8·9	2·3, 34·8	1·2	0·8, 1·8	2·7	0·9, 7·8
Economic status								
5th quintile (richest)†	–	–	–	–	–	–	–	–
4th quintile	1·6	1·2, 2·1	1·6	1·1, 2·3	0·9	0·6, 1·5	1·2	0·6, 2·1
3rd quintile	2·1	1·5, 3·0	2·0	1·2, 3·3	0·8	0·5, 1·4	0·7	0·4, 1·3
2nd quintile	3·0	2·0, 4·3	3·9	2·4, 6·4	1·2	0·8, 2·0	1·1	0·6, 1·9
1st quintile (poorest)	3·9	2·3, 6·8	4·5	1·9, 10·6	2·2	1·4, 3·5	1·6	1·0, 2·8
Household size								
0–4 persons†			–	–	–	–	–	–
5–8 persons			1·8	1·2, 2·7			1·5	1·2, 2·0
>8 persons			1·7	0·6, 4·9			5·2	2·5, 10·8
Language								
Spanish†			–	–	–	–	–	–
Gurani			0·8	0·5, 1·2			0·7	0·4, 1·3
Bilingual			0·7	0·5, 0·9			0·8	0·4, 1·4
Others			0·5	0·1, 2·0			1·9	0·9, 3·7
Male household education level								
None†			–	–	–	–	–	–
Primary			1·6	0·5, 4·7			0·9	0·5, 1·6
Middle			1·7	0·5, 5·6			0·8	0·4, 1·5
Secondary or higher			1·7	0·5, 5·8			0·7	0·3, 1·8
Female household education level								
None†			–	–	–	–	–	–
Primary			0·3	0·1, 1·1			0·6	0·4, 1·0
Middle			0·2	0·1, 0·8			0·6	0·3, 1·0
Secondary or higher			0·2	0·1, 0·9			0·3	0·1, 0·6

* OR estimates and the associated 95 % CI from the multivariate logistic regression of the Nutritional Deprivation Index on household income and other household characteristics, run separately for rural and urban households; rural models were run with 1971 observations and urban models with 3446 observations. See Table Al in the Appendix for variable definitions. Source: authors’ calculations.

† Reference group.
